# Scaling up sexually transmissible infections point-of-care testing in remote Aboriginal and Torres Strait Islander communities: healthcare workers’ perceptions of the barriers and facilitators

**DOI:** 10.1186/s43058-021-00232-8

**Published:** 2021-11-07

**Authors:** Lise Lafferty, Kirsty Smith, Louise Causer, Kelly Andrewartha, David Whiley, Steven G. Badman, Basil Donovan, Lorraine Anderson, Annie Tangey, Donna Mak, Lisa Maher, Mark Shephard, Rebecca Guy, on behalf of the TTANGO2 Collaboration, Lisa Bastian, James Ward, John Kaldor, Crissy Comerford, Trish Bushby, Liz Moore, Manoji Gunathilake, David Johnson, Daniel Gallant

**Affiliations:** 1 The Kirby Institute, UNSW Sydney, Sydney, NSW 2052 Australia; 2 Centre for Social Research in Health, UNSW Sydney, Sydney, NSW 2052 Australia; 3 Flinders University International Centre for Point-of-Care Testing, Flinders University, Adelaide, SA 5042 Australia; 4 University of Queensland, Brisbane, QLD 4006 Australia; 5 Kimberley Aboriginal Medical Services, Broome, WA 6725 Australia; 6 Ngaanyatjarra Health Service, Alice Springs, NT 0870 Australia; 7 Department of Health, Western Australia, East Perth, WA 6004 Australia; 8 School of Medicine, University of Notre Dame Australia, Fremantle, WA 6160 Australia; 9 Burnet Institute, Melbourne, VIC 3004 Australia

**Keywords:** Sexually transmissible infections (STIs), Point of care testing (POCT), Aboriginal and Torres Strait Islander, Acceptability, Scaling up, Qualitative research

## Abstract

**Background:**

Sexually transmissible infections (STIs), such as gonorrhoea and chlamydia, are highly prevalent, particularly in remote Aboriginal and Torres Strait Islander communities in Australia. In these settings, due to distance to centralised laboratories, the return of laboratory test results can take a week or longer, and many young people do not receive treatment, or it is considerably delayed. Point-of-care testing (POCT) provides an opportunity for same day diagnosis and treatment. Molecular POC testing for STIs was available at 31 regional or remote primary health care clinic sites through the Test-Treat-And-GO (TANGO2) program. This qualitative study sought to identify barriers and facilitators to further scaling up STI POCT in remote Aboriginal communities within Australia.

**Methods:**

A total of 15 healthcare workers (including nurses and Aboriginal health practitioners) and five managers (including clinic coordinators and practice managers) were recruited from remote health services involved in the TTANGO2 program to participate in semi-structured in-depth interviews. Health services’ clinics were purposively selected to include those with high or low STI POCT uptake. Personnel participants were selected via a hybrid approach including nomination by clinic managers and purposive sampling to include those in roles relevant to STI testing and treatment and those who had received TTANGO2 training for POCT technology. Milat’s scaling up guide informed the coding framework and analysis.

**Results:**

Acceptability of STI POCT technology among healthcare workers and managers was predominantly influenced by self-efficacy and perceived effectiveness of POCT technology as well as perceptions of additional workload burden associated with POCT. Barriers to integration of STI POCT included retention of trained staff to conduct POCT. Patient reach (including strategies for patient engagement) was broadly considered an enabler for STI testing scale up using POCT technology.

**Conclusions:**

Remote healthcare clinics should be supported by both program and clinic management throughout scaling up efforts to ensure broad acceptability of STI POCT as well as addressing local health systems’ issues and identifying and enhancing opportunities for patient engagement.

**Supplementary Information:**

The online version contains supplementary material available at 10.1186/s43058-021-00232-8.

Contributions to the literature

- Identification of implementation challenges for STI POCT in remote Australia and other settings where laboratory diagnosis is not available, or significant delays occur
- There is broad *acceptability* of STI POCT among healthcare workers in communities with high endemicity of STIs
- Healthcare worker recommendations to improve acceptability, compatibility, and reach in STI POCT scale up
- Identification of healthcare workers’ barriers to POCT use, as well as recommendations to improve POCT operator self-efficacy, such as integration of timely refresher training/s

## Background

The high prevalence of sexually transmissible infections (STIs) in many parts of the world requires effective and immediate test and treat interventions to reduce transmission [1]. Globally, an estimated 131 million cases of genital *Chlamydia trachomatis* (CT), 78 million cases of *Neisseria gonorrhoeae* (NG), and 142 million cases of *Trichomonas vaginalis* (TV) infection are reported every year among people aged 15–49 years [1]. Within Australia, Aboriginal and Torres Strait Islander peoples are disproportionately diagnosed with CT, NG, and TV infections compared with the non-Aboriginal population [2, 3], requiring urgent action in STI response and management to achieve greater health equity. All three STIs are easily treatable, but can have serious acute and chronic health consequences if left untreated, including reproductive complications for women (pelvic inflammatory disease, infertility, and ectopic pregnancy) [4, 5], increased risk of HIV [6], along with psychosocial factors such as shame and embarrassment [7, 8]. Untreated STIs in pregnancy can have consequences for the newborn, such as prematurity, low birth weight, and neonatal morbidity [9].

Considering these infections are mostly asymptomatic, testing is key to identification and prompt treatment. Many resource-limited countries rely on syndromic management which is inaccurate and leaves most infections undiagnosed (and untreated) [10]. In high-income countries, laboratory-based testing is available, but there are significant delays within many remote communities to receive results, hindering timely treatment and public health responses [11]. In these settings, point-of-care testing (POCT) may be advantageous for etiological diagnosis [12, 13]. In 2013, the GeneXpert CT/NG assay (Cepheid, Sunnydale, California, USA)—a molecular-based technology conducted on the GeneXpert testing platform—became available for use in Australia, enabling accurate STI testing at or near the point-of-care. The GeneXpert CT/NG assay has been shown to accurately detect CT and NG in a variety of asymptomatic populations in several low- and middle-income countries (e.g. South Africa [14], Swaziland [15], Tanzania [16], and Papua New Guinea [17]) and to be effective for identification of asymptomatic STIs among high-risk populations [18]. Other molecular-based STI point-of-care tests available or in the pipeline may offer shorter turnaround times (< 60 min, including GeneXpert CT/NG and TV assays), multiplex testing (STI arrays), and smaller, more portable testing devices; however, their operational performance and clinical impact are yet to be determined. The particular advantage of the GeneXpert system is the ability to test not only for CT/NG and TV but also for other infectious diseases of concern including HIV, HCV, and HPV using specific assay cartridges run on the same device [19]. To increase supply and reduce costs of available tests, the World Health Organization (WHO) released a target product profile in 2014 to communicate with industry the type of point-of-care testing (POCT) technology needed [20]; with a second WHO model list of essential diagnostics released in 2019 [21]. Due to the advancing technology of STI POCT, and the ongoing epidemic of treatable STIs, there has been a recent call to action for health systems integration of STI POCT [22].

The Test Treat ANd GO (TTANGO) trial, conducted in Australian Aboriginal and Torres Strait Islander communities, represented the world-first implementation of POCT for CT/NG at the primary care level [23]. This randomised control trial (2013–2015) used the GeneXpert CT/NG assay performed by clinicians at the point-of-care and demonstrated that use of POCT significantly increased treatment uptake and reduced time to treatment [24]. The operational performance of the GeneXpert CT/NG test conducted at the point-of-care by trained primary health care workers was highly concordant with conventional reference laboratory-based CT/NG testing [25]. In-depth interviews showed healthcare worker confidence in POCT results, perceived improvements in the management of STIs, and greater job satisfaction [26]. Since 2016, POCT with the GeneXpert CT/NG assay has been translated and expanded into a routine program (TTANGO2) [27] with the addition of the TV test at participating remote primary health care services across four jurisdictions (Northern Territory, Queensland, South Australia, and Western Australia,). During the trial, the GeneXpert, associated medical equipment (cartridges, pipettes, etc.), training (including access to help desk support), quality management program, and digital connectivity were provided to each of the study sites free of charge as part of a time-limited program. With support of additional funding, the next phase of the program (TTANGO3) will involve expansion to 45 health services in total by mid-2021.

Healthcare interventions shown to be effective, such as CT/NG POCT, should be assessed for both scale up and scalability [28–30]. According to the WHO, scaling up is the “deliberate efforts to increase the impact of successfully tested health innovations so as to benefit more people and to foster policy and programme development on a lasting basis” [2, 31]. Scalability, or “spread”, is the ability to widely implement an intervention [29, 32]. Understanding the barriers and enablers to STI POCT implementation, use, and health service integration is necessary to support successful scaling up in remote settings. Acceptability of healthcare interventions encompasses a multi-faceted construct of seven components: affective attitude, burden, ethicality, intervention coherence, opportunity costs, perceived effectiveness, and self-efficacy [33]. Using Milat et al.’s scaling up guide [28] in conjunction with Sekhon et al.’s acceptability framework [33], this paper seeks to identify the barriers and facilitators of scaling up STI POCT in high prevalence settings. Combined, these constructs inform the degree to which a healthcare intervention is deemed acceptable by those delivering, or those receiving, the intervention (i.e. healthcare workers delivering STI POCT).

The initial TTANGO trial showed broad acceptability of STI POCT in Aboriginal and Torres Strait Islander communities but was conducted in the context of a formal research trial [26]. Our study described here, conducted in the context of the translated routine STI POCT program (TTANGO2), explores in more depth, the barriers inhibiting, and facilitators enabling full scale up of STI POCT in the real-world setting of remote Aboriginal and Torres Strait Islander communities. It is within this context we investigated the acceptability of STI POCT and explored potential influences on full scale up of POCT technology.

## Methods

The primary clinics participating in TTANGO2 were located in remote areas of Australia across four jurisdictions: South Australia, Western Australia, the Northern Territory, and Queensland. These clinics predominantly provide care to Aboriginal and Torres Strait Islander people in small, often isolated, remote communities. Services were mostly Aboriginal Community Controlled Health Services (ACCHS) with some services managed by jurisdictional health departments. Remote Health clinics vary in size and resources—many smaller remote health clinics are staffed by two Remote Area Nurses and/or Aboriginal Health Practitioners providing emergency care 24/7 along with chronic condition management and preventative care, and others include broader staffing with receptionists, allied health professionals and supported by public health programs.

All services had a community population size of > 500 and were eligible for participation in the TTANGO2 program because they met the criteria of offering CT/NG testing to > 150 young people annually and a positivity of > 10% CT/NG among 16 to 29 year old [23]. For this qualitative evaluation, clinics were purposefully selected to include representation of ‘high’ (*n* = 3 clinics) or ‘low’ (*n* = 4 clinics) POCT sites. Sites were deemed ‘high’ testing clinics if they had conducted more than 150 point-of-care tests over the previous year *and* consistently reported > 10 point-of-care tests per month. Sites were considered ‘low’ testing clinics if they conducted fewer than 150 point-of-care tests over the previous year *and* consistently reported 10 or fewer POCT per month.

Assessing the clinics involved in this qualitative component as either ‘high’ or ‘low’ performing sites provided an understanding of the barriers and facilitators experienced by healthcare workers and managers in integrating STI POCT into their service. It must be acknowledged that assessment of clinics on a single domain—use of STI POCT—is not indicative of the service’s performance in other domains [34]; thus services deemed to be ‘low performing’ by our metric may be high performing in other areas, such as patient engagement, allied health, or other clinical care. Additionally, testing numbers may not accurately reflect the proportion of patients accessing the service, but rather, are indicative of the frequency of use of POCT technology within the clinic which may have bearing on integration into standard care. See Table 1 for a summary of POCT numbers across the clinics included. Six clinics were classified by the Australian Bureau of Statistics [35] as very remote and one as remote, hereafter referred to as remote.

**Table 1:** POCT by clinic

Clinic	Total # of POC tests (preceding 12 months)	High/low performance
Clinic A	75	Low
Clinic B	89	Low
Clinic C	66	Low
Clinic D	28	Low
Clinic E	618	High
Clinic F	173	High
Clinic G	173	High

Using purposive sampling [36], we sought to interview comparable personnel from each of the selected clinics who worked in positions relevant to sexual health and had received TTANGO2 training for operating POCT technology. Practice managers/clinic coordinators were contacted by a member of the study team seeking support for clinic personnel participation and suggested participants. The interviewer (LL) then reached out to personnel directly, either in person (two clinics) or via phone/email. One nurse declined to participate; a reason was not provided. Participants’ responses are noted as belonging to an interviewee from a ‘high’ or ‘low’ STI POCT clinic (as defined above); to ensure anonymity, service coordinators and practice/clinic managers are listed as ‘management’.

All participants were provided with a participant information statement prior to obtaining signed consent. Interviews were conducted in person (two clinics; nine interviews) and via phone (five clinics; 11 interviews). The median duration of interviews was 29 min. Participants were not remunerated. Interviews were audio-recorded and transcribed verbatim by a professional transcriber. Transcripts were proofed for accuracy and de-identified.

The interview guide was developed among the study authors and was reviewed by both the TTANGO2 Investigator Group and Executive Group; comments were integrated prior to data collection. Topics included questions around the use of STI POCT, specifically relating to feasibility and sustainability, normalisation of STI POCT/healthcare delivery experiences, community and cultural acceptability, evaluation and sustainability, and demographic characteristics of participants (age, gender, length of time in remote health sector, current position, and time in current role) and was reviewed by all co-authors. The study methods and results adhere to the Consolidated Criteria for Reporting Qualitative Research (COREQ) [37]; see Supplementary Material: SM1 COREQ checklist.

De-identified transcripts were uploaded to NVivo qualitative software (version 12). The first round of deductive coding was informed by the interview guide [38]. A second round of iterative coding was conducted to identify barriers and facilitators to scaling up using Milat et al.’s [28]. “Increasing the scale of population health interventions: A Guide”, inclusive of acceptability. To better interrogate the data regarding acceptability within a scaling up framework, we have utilised Sekhon and colleagues’ [33] seven component constructs of ‘Acceptability’ of healthcare interventions (affective attitude, burden, perceived effectiveness, ethicality, intervention coherence, opportunity costs, and self-efficacy), within the second round of coding. Underpinned by a latent thematic analysis approach [39], themes were synthesised within each of these nodes. This analytic approach enabled observation of the ways in which themes complemented and overlapped within the scale-up and acceptability frameworks. Definitions of the components, along with our interpretations and application to the data, are presented in Table 2.

**Table 2:** Definitions and interpretations of acceptability and scale-up components

Component	Definition	Interpretation/Application
Acceptability [1]		
Burden	“the perceived amount of effort that is required to participate in the intervention”	Depictions of workload burden associated with STI POCT
Self-efficacy	“The participants confidence that they can perform the behaviour(s) required to participate in the intervention”	Competence/confidence in operating and implementing STI POCT
Perceived effectiveness	“The extent to which the intervention is perceived as likely to achieve it’s purpose”	Effectiveness of STI POCT compared with standard pathology/existing STI test-and-treat pathways
Scale up [2]		
Compatibility	“how well the intervention fits with the systems, services and practices of the new environment or setting”	Broader health systems, operations, and clinics within which STI POCT is being implemented
Reach	“the level of contact with or individual participation of an intended target population in an intervention”	Patient engagement for implementation of STI POCT

This qualitative analysis focuses on the experiences of the healthcare workforce within the contexts of acceptability and scale up of STI POCT. However, we have included patient reach from the perspective of health care workers, as this was consistently considered an important component of consideration for scale up efforts. Following preliminary analysis, co-authors were consulted regarding data interpretation among their relevant area(s) of expertise (i.e., pathology/point-of-care technology, Aboriginal health, sexual health) to ensure interpretation within specialised context.

Ethics approvals for this qualitative study were obtained from the Aboriginal Health Research and Ethics Committee (HREC 04-15-626) (South Australia), Aboriginal Health Council of Western Australia (Project 644), Central Australian Human Research Ethics Committee (Project 16-373) (Northern Territory), and Far North Queensland Human Research Ethics Committee (HREC/15/QCH/66—986).

## Results

Interviews were undertaken with a range of healthcare workers including Aboriginal Health Practitioners (AHP) (*n* = 8), Registered Nurses (RN) (including Remote Areas Nurses and Clinical Nurse Consultants) (*n* = 7), service coordinators (including sexual health and chronic disease) (*n* = 2), and practice/clinic managers (*n* = 3).

### Acceptability

Acceptability is defined as the “the degree of support for the intervention among stakeholders” [2, 28] and was broadly indicated among RNs, AHPs, and managers for STI POCT. However, there were some contributing factors to participants’ overall acceptability of the POCT device, including burden, self-efficacy, and perceived effectiveness.

#### Burden

Burden, referring to “the perceived amount of effort that is required to participate in the intervention” [8, 33], was raised by some participants. Perceptions of burden of the intervention varied across healthcare settings, with those from high testing clinics indicating ease of use of the device and timeliness of results as reducing workload. Having a large proportion of staff who were trained to use the device was seen as beneficial in sharing the potential workload, enabling clinicians (such as General Practitioners) to pass on samples to be tested by trained operators. Having multiple trained operators also aided time management within a clinic, with trained personnel able to progress the flow of POCT rather than a possible delay if reliant on a single test operator. Importantly, the size of the clinic (or total number of personnel) was not a factor in determining whether POCT was integrated into service. Instead, the key consideration was that participants had been trained in POCT *and* implemented this into their work practice.

Everyone gets trained. It’s really hard when there’s only one or two people who can do it to start with and then if one wants you to go … then that’s where it gets time consuming when everyone wants you to do the [STI POCT] and you’re the only person that’s trained in the clinic. So you need to have everyone trained, otherwise, it’s too hard. (High POCT Clinic, Nurse)

We do fairly low numbers at the moment. [*Okay, why is that?*] Hmm … Because I’m the only operator, so it has to be me that does them. […] So I need to either see someone is doing an STI check on someone and say, “Wait, give us a sample, and I’ll go and do [a point-of-care test] for you,” if I can get a chance to do that. Maybe sometimes it’s not appropriate I stick my face in [to the consult room] when the patient’s sitting there. (Low POCT Clinic, Nurse)

#### Self-efficacy

Self-efficacy refers to “the participant’s confidence that they can perform the behaviour(s) required to participate in the intervention” [8, 33]. STI POCT often was not prioritised in clinics with high patient loads, reduced staffing, or other factors which contribute to busyness and workload. However, it is likely that unfamiliarity with the device and performing a test, which may take more time than expected for those who do not use the technology regularly, act as a barrier during busier clinic hours. Several participants suggested that frequent or regular use of POCT would assist in overcoming this barrier as healthcare workers became more familiar with the process. By contrast, high testing clinic staff viewed the machine as “easy” once its use was regularly integrated into practice, whereas low testing clinics described integration as a future action to be taken. Infrequent use of POCT resulted in reduced confidence in performing POCT, thereby creating an additional barrier, or burden, for healthcare workers.

It’s just one of those things, if you don’t use it like regularly every single day, you have to really stop and think of, you know, the steps. (Low POCT Clinic, Management)

It’s just a matter of getting used to the system and how it works. Once you’ve got it … once you start doing it, it’s very easy. (High POCT Clinic, Nurse)

However, several participants suggested refresher training to develop self-efficacy to perform POCT, particularly among healthcare workers not regularly using POCT following the initial training. The proposal of refresher courses was viewed as an opportunity to regain confidence and assist in timeliness for upskilling new employees. It should be noted that refresher training is available to operators on an as-needs basis via the Help Desk; however, the findings suggest that this could be made better known as many operators did not seem to be aware of this service. Additionally, refresher training is designed into the program, with the requirement for all certified POCT operators to complete refresher training every 2 years at the expiry of their competency.

Just making sure that you have all staff trained and continually giving them updates and refreshers because, as I said, if you don’t use it every single day and then you, I suppose, like a week or two weeks later, put a specimen through, you really have to think, “okay, alright, have I done that step, done that step”, but if you were keeping your staff refreshed with training all the time, it wouldn’t be that … they wouldn’t have to think about it. They’d just basically go in and do it. (Low POCT Clinic, Management)

Yeah for everybody, because we always have new staff as well. Do it like not one on one, because I think it’s a waste of time when funding just brings that trainer here to train one person. […] Also I think it’s a good refresher for those people that are trained, maybe they don’t feel confident yet, but they feel like they want to refresh it? (Low POCT Clinic, Nurse)

Significant barriers to increased scaling up of STI POCT that were identified by participants included physical hardware (including a molecular testing device, cartridge scanner, laptop, cables, and in some cases, a USB Wi-Fi stick), computer literacy (using a laptop, Windows Operating system and layered software to generate digital test orders—e.g. ONDAS (Clinical Universe, Adelaide, SA AUSTRALIA)), and performing the test—i.e. GeneXpert Dx (Cepheid, Sunnyvale, CA USA). Several participants indicated the testing process is “not user friendly”. Other participants described the dual software as not being “intuitive” through its differing layout to the standard patient management software (and data entry prompts—e.g. input order of first or surname) used in the primary care clinics. These issues were raised by personnel from both high and low testing clinics as increasing burden and reducing self-efficacy.

You’re using Communicare as your …, and everything’s different on that to the interface on the [GeneXpert] laptop and all that, it’s just that you keep putting your brain in another place and when you’re really busy, I often hear staff going […] “What’s happened here? Why is this screen up? This isn’t what I asked for,” and I hear that sort of frustration come through. (High POCT Clinic, Management)

[Operating the POCT technology] does get a bit confusing … if you haven’t been doing it regularly, you can just … it kind of slides out away from you. (Low POCT Clinic, Management)

#### Perceived effectiveness

Perceived effectiveness is the “extent to which the intervention is perceived as likely to achieve its purpose” [8, 33]. The effectiveness, or purpose of STI POCT, is to ensure timely treatment via rapid testing and diagnosis. POCT allows for same-day treatment, which, in combination with increasing testing coverage, has been shown in mathematical modelling to have the potential to reduce community STI incidence and prevalence in remote Aboriginal and Torres Strait Islander communities [40]. This was widely viewed as beneficial among the participants involved in this qualitative study with several participants (among both high and low testing clinics) commenting on the possibility of STI POCT and treatment as halting or reducing the transmission of STIs, particularly in high endemic, and highly transient settings, such as some remote Aboriginal and Torres Strait Islander communities.

I would highly recommend [POCT] as a tool for STI [testing and treatment] because [of] the rate that those things can spread within a week. Someone who’s infected can spread that stuff around several times within a week and then you’re chasing, you know, the mathematics just come in then, you know. If four people get infected in one week, and then they go see three more people each, and then those three people see two more people. […] [STI POCT is] an invaluable tool to nip [STI prevalence] in the bud quickly. (High POCT Clinic, Nurse)

### Compatibility

Compatibility refers to “how well the intervention fits with the systems, services and practices of the new environment or setting” [WHO, 2010, cited in: 28:2]. Broadly, participants indicated that STI POCT fit well within the current health services and practices, describing the intervention as “a very important tool of our trade” (High POCT Clinic, Nurse). At high testing clinics, training healthcare personnel on STIs was suggested as a means for better tailoring STI POCT within the systems, services, and practices of remote healthcare clinics. Locum nurses, who are often recruited on short-term contracts (often ranging from two weeks to a few months) to work in remote healthcare clinics [41], were viewed as a barrier to scaling up STI POCT due to their lack of knowledge relating to the high prevalence of STIs in the communities to which they are contracted.

I guess another thing is that, you know, staff that are recruited that work remotely are emergency trained staff, so they’re not primary healthcare trained on average, and so certainly not sexual health, so if you work in emergency and intensive care, sexual health is not even on your radar. So, most staff know nothing about sexual health. I mean […] because this is my job, so I train staff to it, but I guess when you look at a lot of other organisations, there’s no orientation to sexual health in particular, so I think staff are not aware of the burden of disease that we have. So I guess if someone was symptomatic, they’d think, “Oh, gosh, what could that be?” but the fact that the majority of people are asymptomatic and the burden is so high is possibly not on their radar. (High POCT Clinic, Management)

The remoteness of the clinics, and distance to reference laboratories (with time delays in specimen transfer and follow up with patients to deliver results and treatment as required), made STI POCT highly compatible for reducing STI disease burden in remote communities, granting services autonomy to rapidly diagnose and manage STIs, particularly with regards to the scalability of POCT. The use of POCT was viewed as significantly shortening the wait time for test results and aiding in opportunities for timely treatment.

The isolation, the time it takes for the results to come back, I think that would be a critical thing to consider in using it. […] If you can get results the next day or you’re really close to pathology, you probably don’t need it but, you know, most of these communities are hundreds of kilometres away from anyone and it’s only planes and planes aren’t always going. (High POCT Clinic, Nurse)

POCT was viewed as highly compatible within clinics as it enabled faster response time for patient follow up, thereby increasing likelihood of locating the patient in the community (as most patients did not wait around for results) and providing same-day treatment.

So it’s nice to know straight away or, you know, within an hour or two, and you can treat appropriately. (High POCT Clinic, Nurse)

The lab will take about, as I said, five to seven days, so, you know, with the [GeneXpert] within say 90 minutes, we’re able to record and treat, you know, rather than waiting. (High POCT Clinic, Nurse)

To get the result in about an hour and a half means we can do it in the morning [or follow up if needed, then] treat them [in the afternoon] if you haven’t treated them already and get things rolling in terms of the long-term outcomes for that patient. And you can start doing things like your contact tracings and stuff much sooner because you know you’ve got a positive result. (Low POCT Clinic, Nurse)

### Reach

Reach has been defined as “the level of contact with or individual participation of an intended target population in an intervention” [Glasgow, Vogt, & Boles, 1999, cited in: 28:2]. In this regard, patient engagement was viewed as multifactorial in the effectiveness of STI POCT scale up. Education of patients was viewed as necessary with some healthcare workers highlighting a lack of patient awareness that treatment is available for certain STIs and that both testing and treatment can be delivered quickly when using POCT technology. Prompt results were believed to alleviate patient anxiety. There was recognition that communication, inclusive of verbal and non-verbal, as well as patient-healthcare provider rapport, are instrumental for patient engagement, particularly in allaying feelings of shame regarding sexual behaviours and associated risk practices (such as sex without a condom). Inclusion of strategies for patient engagement within STI POCT training was viewed as a strategy to assist with enhancing patient reach and ultimately increasing the volume of STI POCT.

[B]ecause the clients don’t even know there’s a machine that can test for gonorrhoea and chlamydia, and that test only takes 90 minutes. So like I reckon we’ve got to put posters up around the clinics and like advertise it a bit more, so people can say, “Oh, wow! We can just get it … ask the nurse if we can do a screening on us and do the [POCT].” (Low POCT Clinic, AHP)

I tend to say to [patients] when I see people in the clinic, it’s actually too long, you know, and I just say, “Where are you going to be this afternoon? What house will you be at? If I come looking for you in the next couple of hours, you know, I’ll come to that house […].” And I find that they are quite interested in that idea and are like, “Oh, we’re going to find out the same day.” (High POCT Clinic, Management)

Overlapping with patient education was the role of communication as a way for reducing stigma around sexual risk practices and sexual health while normalising STI screening.

Well, there is a lot of people who are ashamed, you know, just to get a test done like they’re shy to come in or what not, but just informing them that it’s nothing, we’re not saying that you do have any sexual STIs or anything, but just to get it checked like, it’s just normal like to get screened every once in a while. (Low POCT Clinic, AHP)

I mean people just come in to have a dressing, offer [STI POCT] and explain that you test for everything. I mean it’s not only your reproductive system, it’s also your diabetes or whatever, your kidney, but you just have to learn how to sell it, that’s all. […] I’m not trying to stigmatise your health for STI, but just make it a bit broader, bit of holistic approach, I suppose. (High POCT Clinic, AHP)

## Discussion

Focusing on the perspectives of healthcare workers and utilising Milat’s scale up framework, three main themes emerged as instrumental in scale up efforts with barriers and facilitators identified across both: acceptability (including burden, perceived effectiveness, and self-efficacy), compatibility, and reach. Combined, these key issues illustrate the nuanced barriers and facilitators to STI POCT scale up in communities where distance to standard pathology or other hindrances exist. Commonalities existed across both high and low testing clinics, including perceptions that STI POCT was beneficial for reducing prevalence, and suggestions that the IT components could be more “user friendly”. Interestingly, refresher training emerged as a strategy for improving self-efficacy among low testing clinics (as it was viewed refresher training would increase confidence), while STI training was viewed among those in high testing clinics as an important educational piece for locum workers within the context of compatibility. Nonetheless, increased learning opportunities were seen as a favourable strategy for scale up efforts of STI POCT.

Burden, as a construct of acceptability, was minimised in high testing clinics through having a large proportion of the healthcare workforce trained as POCT operators. Training personnel to use POCT technology is often overlooked as a priority by the clinic, leaving the testing burden to fall to a small number of staff. In a study exploring healthcare workers’ acceptability of syphilis POCT in Zambia, healthcare workers reported increased burden associated with integrating POCT, particularly for those not previously involved in syphilis POCT and where staffing was an issue [42]. Our findings suggest that greater POCT training among the healthcare workforce (particularly among transient/locum staff) would likely reduce workload burden as responsibilities for testing can be shared among healthcare workers rather than relying on a small number of personnel to conduct STI screening. However, this would need to be balanced against the training requirements for molecular POC testing, which are greater than other simpler lateral flow tests. Participants identified two skill sets necessary for healthcare workers to increase STI POCT: (1) knowing when, how, and why to offer opportunistic testing and (2) how to operate the STI POCT technology. It has been recommended that healthcare settings which are integrating POCT technology should increase their workforce as a means to reduce increased burden on the existing personnel and the unintended consequence of “bottlenecks” in clinic operations and workflow [22, 23, 43].

Regular and frequent use (and optimisation) of POCT technology likely increased self-efficacy of conducting STI POCT. Developing mindlessness requires repetition—whereby an act becomes so routine it requires little cognitive thought to be completed [44]— and may reduce barriers of self-efficacy as confidence in using POCT technology becomes habituated. Clinics which had integrated STI POCT into routine practice reported higher self-efficacy and reduced burden in using POCT. Other research has suggested that STI screening can be normalised through the integration of STI testing into annual adult health checks as standard practice, particularly among young men who miss out on reproductive health opportunities (i.e. pregnancy) to be offered STI testing [45]. This normalisation would likely provide dual benefits with more hands-on experience with the POCT technology (leading to greater self-efficacy) and increasing testing of patients (leading to better STI management). Notwithstanding the barriers noted across all domains, healthcare workers have performed almost 34,000 STI point-of-care tests to March 2021.

Familiarity, confidence, and ease-of-use of the POC testing equipment and information technology appeared to impact self-efficacy. The connectivity system implemented for TTANGO2 required the use of two software programs to allow for return of digital test results in keeping with usual practices in these clinics. The proprietary GeneXpert software available was not sufficient to meet requirements for local digital results integration as was preferred by clinics. The addition of a second piece of software, ONDAS, was needed to allow for sufficient patient information to be captured at the time of testing, to minimise risk of data entry errors and ensure POC test results would be delivered to the patient management system and matched with the corresponding electronic patient record. Improvement in the user interface and simplification of the user experience are important to enable infrequent users to perform a test and not find navigation of such technology a barrier. A new user GeneXpert interface, called Xpress, is now available and more user friendly, i.e. touch screen tablet, but this is not yet compatible for STI POCT [46]. It should be noted that the GeneXpert has two types of software: GX DX and Xpress. The Xpress is not yet available for STI POCT (currently only for COVID and Flu A/B/respiratory syncytial virus) [46] and another forthcoming Xpress CT/NG test which will provide results within 30 minutes with simplified software. Limitations of the systems available (as used within TTANGO2) include use of the two-layered system as DX alone could not capture enough data to ensure data quality and the key identifiers to allow for return of electronic results to the patient management software that the clinics wanted as a priority. To the authors' knowledge, the Xpress at present also cannot do this. Also, clinics that use only the DX have made errors in entry of patient information as there are no field checks or balances or ways to prepopulate information during implementation of the TTANGO2 program. The Xpress version may not fully resolve the gaps in connectivity issues with earlier versions to ensure digital integration and other benefits of connectivity in the context of a decentralised POC testing program implemented in remote Australian primary health care settings. As the platform for molecular POCT enables on-site POCT for SARS-CoV-2 [47], RSV, Flu A, B and other infectious diseases such as hepatitis C and HPV, these findings are applicable to scale up of POCT (beyond STIs) in these remote settings and other primary care settings in Australia and remote settings elsewhere.

Refresher training was recommended by personnel as a mechanism to build and regain confidence among people who may not have used POCT technology frequently since the initial training or have had time away from the clinic. The TTANGO2 program routinely provides refresher training to operators every two years at competency expiry. Refresher training is also available to operators on an as-needs basis; however, based on the findings, this was not commonly known among operators. More frequent refresher training would be beneficial to promote self-efficacy and support integration of routine use of POCT. Healthcare workers delivering syphilis POCT in Ghana identified a lack of refresher training as a barrier to rollout [48]. In a study focused on nurses receiving glucose POCT training, it was found that a single training was “inefficient” and that refresher training was necessary to achieve proficiency [49]. Other research has shown that refresher trainings increased healthcare worker proficiency of syphilis POCT, as well as enabling refresher training attendees to upskill their colleagues following course completion [50]. Similarly, refresher training for laboratory personnel has been recommended by the WHO “to maintain their expertise” as a pre-outbreak preparedness strategy [1, 51], suggesting that refresher trainings are beneficial for skilled laboratory technicians as well as healthcare workers who may be new to conducting and running POCT.

The availability of STI testing and results at the point-of-care indicated strong compatibility of STI POCT among high performing clinics. Similar to our findings, distance to laboratory, and associated lengthy wait times for test results, have previously been identified as a barrier to STI management in remote Aboriginal and Torres Strait Islander communities [52], suggesting that STI POCT is highly compatible within remote healthcare delivery settings. Indicative within our research was the need for locum nurses working in the remote health sector to receive necessary education (including understanding the importance of opportunistic testing and strategies for patient engagement, as well as cultural safety) to equip them to respond to the local needs of the communities in which they work—a response that has been called upon within the literature [53].

Reach of patients encompassed education and communication as necessary aspects for effective patient engagement through informing patients about STI testing and availability of treatment, as well as reducing stigma associated with STI screening. STI POCT has been attributed as being an important tool for reducing stigma and “making testing a normative healthcare behaviour” [2, 54].

Our study has some limitations. While findings present a range of perspectives about STI POCT scale up from AHPs, RNs, and coordinators/managers in seven health clinics involved in the TTANGO2 program, clinics were selected on the basis of high/low POCT uptake. Participants recruited from these clinics may have utilised the GeneXpert more or less than their colleagues within individual clinics—thus, responses may be indicative of personal experience rather than broader experiences within clinics. Additionally, the cultural and contextual experiences of Aboriginal and Torres Strait Islander communities can vary widely [55]; thus, what may be regarded as a barrier or facilitator to STI patient engagement in one community may not have relevance in another. Consequently, the findings presented here are indicative of the experiences of the individual personnel and communities from which the data were collected and are not generalisable to all staff or all health clinics/services in remote communities. However, these results provide insights into the unique challenges and enablers which may occur in remote health settings regarding STI POCT and should be considered in scale up efforts in this context.

## Conclusions

While there is strong acceptability of STI POCT among healthcare workers in regional and remote Aboriginal and Torres Strait Islander communities in the participating study sites, there remain some barriers to full scale up of POCT. In remote communities, staff turnover remains an ongoing barrier to increased STI POCT, and uptake/integration of POCT use. Scale up efforts should seek to communicate the benefits of STI POCT to healthcare workers, and address challenges and barriers early, such as providing opportunities within clinics for trained healthcare workers to increase self-efficacy in using POCT technology (either through regular use or refresher training). This would have the additional benefit of ensuring the burden of POCT is better distributed among employees rather than falling to one or two workers within clinics. Furthermore, strategies to increase patient and community information about STI POCT, and to reduce shame and stigma associated with sexual health more generally, should underpin any scale up efforts.

## Supplementary Information

**Additional file 1.**

## Data Availability

The data that support the findings of this study are not publicly available as they contained information that could compromise research participant privacy/consent.

## Abbreviations

ACCHS: Aboriginal Community Controlled Health Services
AHP: Aboriginal Health Practitioner
CT: Chlamydia
NG: Gonorrhoea
POCT: Point-of-care testing
RN: Registered Nurse
STI: Sexually transmissible infections
TTANGO: Test Treat ANd GO
TV: Trichomonas vaginalis
