# Supplementary material for: Scaling up sexually transmissible infections point-of-care testing in remote Aboriginal and Torres Strait Islander communities: healthcare workers’ perceptions of the barriers and facilitators
Source: Implement Sci Commun. 2021 Nov 7;2:127. doi: 10.1186/s43058-021-00232-8 (PMC8572571; doi:10.1186/s43058-021-00232-8)
Supplement: Supplementary file 1 — Additional file 1. [file 43058_2021_232_MOESM1_ESM.docx]

COREQ Checklist – Lafferty et al, “Scaling up sexually transmissible infections point-of-care testing in remote Aboriginal and Torres Strait Islander communities: healthcare workers’ perceptions of the barriers and facilitators”

| **No. Item** | **Guide questions/description** | **Response** | **Reported on Page #** |
| --- | --- | --- | --- |
| **Domain 1: Research team and reﬂexivity** |  |  |  |
| *Personal Characteristics* |  |  |  |
| 1. Inter viewer/facilitator | Which author/s conducted the interview or focus group? | Lise Lafferty conducted all interviews | Methods  (Page 9) |
| 2. Credentials | What were the researcher’s credentials? E.g. PhD, MD | PhD, MA (Indigenous Studies), MA (Social Development), BA (Social Science) | N/A |
| 3. Occupation | What was their occupation at the time of the study? | Research Fellow | N/A |
| 4. Gender | Was the researcher male or female? | Female | N/A |
| 5. Experience and training | What experience or training did the researcher have? | Dr Lafferty is a social scientist (BA Social Science, SNHU (USA), 2009) with expertise in Aboriginal and Torres Strait Islander Health (Master of Indigenous Studies, UNSW Sydney, 2011) and social health (PhD, The Kirby Institute, USNW Sydney, 2017). | N/A |
| *Relationship with participants* |  |  |  |
| 6. Relationship established | Was a relationship established prior to study commencement? | All participants had an established relationship with the TTANGO2 program prior to qualitative data collection. Dr Lafferty met participants at the time of interview, with some interaction via phone prior to interview participation. | Methods  (Page 9) |
| 7. Participant knowledge of the interviewer | What did the participants know about the researcher? e.g. personal goals, reasons for doing the research | 7. The Participation Information and Consent Form provided the following information:  “You are invited to participate in an evaluation investigating the feasibility and sustainability of using point-of-care testing for chlamydia and gonorrhoea in healthcare delivery in remote Aboriginal communities. You have been selected as a possible participant because you are employed at one of the health centres in which this study is being conducted. […]  We hope to better understand the barriers and facilitators to sustained provision of STI point-of-care testing and treatment and the community and cultural acceptability of STI point-of-care testing. We hope that this information will enable us to better support other health centres which may start using STI point-of-care testing.  This research involves an interview with a social health researcher.” | N/A |
| 8. Interviewer characteristics | What characteristics were reported about the inter viewer/facilitator? e.g. Bias, assumptions, reasons and interests in the research topic | The interviewer explained to all participants that she did not have a background in pathology or patient care and had undergone the training for using the point-of-care testing technology which all participants had also completed. Additionally, all participants were informed that their response were confidential with the researcher, and that their individual responses would not be reported back to the study team in a way that may individually identify them (their confidentiality was assured). Participants understood that TTANGO2 sought to conduct an evaluation of the program. Potential participants were given opportunity to decline to participate in the interview, or to not provide an answer to any questions asked and a reason for not answering would not be sought. | N/A |
| **Domain 2: study design** |  |  |  |
| *Theoretical framework* |  |  |  |
| 9. Methodological orientation and Theory | What methodological orientation was stated to underpin the study? e.g. grounded theory, discourse analysis, ethnography, phenomenology, content analysis | The analysis was informed by Milat et al’s scale up guide in conjunction with Sekhon et al’s acceptability framework | Methods  (Pages 9-10) |
| *Participant selection* |  |  |  |
| 10. Sampling | How were participants selected? e.g. purposive, convenience, consecutive, snowball | Criterion sampling | Methods  (Page 8) |
| 11. Method of approach | How were participants approached? e.g. face-to-face, telephone, mail, email | Potential participants were approached by the interview either face-to-face (at two clinics) or via telephone (at five clinics) | Methods  (Page 9) |
| 12. Sample size | How many participants were in the study? | 20 | Methods  (Page 9) |
| 13. Non-participation | How many people refused to participate or dropped out? Reasons? | 1 person refused | Methods  (Page 9) |
| *Setting* |  |  |  |
| 14. Setting of data collection | Where was the data collected? e.g. home, clinic, workplace | Interviews were conducted on-site at healthcare clinics (two clinics) and via telephone (five clinics) | Methods  (Page 9) |
| 15. Presence of non-participants | Was anyone else present besides the participants and researchers? | No | N/A |
| 16. Description of sample | What are the important characteristics of the sample? e.g. demographic data, date | Aboriginal Health Practitioners (AHP) (n=8), Registered Nurses (RN) (including Remote Areas Nurses and Clinical Nurse Consultants) (n=7), service coordinators (including sexual health and chronic disease) (n=2), and practice/clinic managers (n=3) | Methods  (Page 9) |
| *Data collection* |  |  |  |
| 17. Interview guide | Were questions, prompts, guides provided by the authors? Was it pilot tested? | Interview guide was prepared, and it was rigorously discussed among the authors. Probes were used to facilitate discussions. | Methods  (Page 9) |
| 18. Repeat interviews | Were repeat interviews carried out? If yes, how many? | No | N/A |
| 19. Audio/visual recording | Did the research use audio or visual recording to collect the data? | Interviews were audio recorded. | Methods (Page 9) |
| 20. Field notes | Were ﬁeld notes made during and/or after the interview or focus group? | Field notes were made after interviews. | N/A |
| 21. Duration | What was the duration of the inter views or focus group? | The mediation duration of the interviews was 29 minutes. | Methods (Page 9) |
| 22. Data saturation | Was data saturation discussed? | The data were collected until similar response were coming following subsequent interviews. We found repeated findings from the responses of 23 participants and did not increase the number of interviews to ensure we did not overburden healthcare workers in high patient-load clinics. | N/A |
| 23. Transcripts returned | Were transcripts returned to participants for comment and/or correction? | No | N/A |
| **Domain 3: analysis and ﬁndings** |  |  |  |
| *Data analysis* |  |  |  |
| 24. Number of data coders | How many data coders coded the data? | One (LL) | N/A |
| 25. Description of the coding tree | Did authors provide a description of the coding tree? | The coding framework was developed among several co-authors. | N/A |
| 26. Derivation of themes | Were themes identiﬁed in advance or derived from the data? | Themes were derived from the data. However, the main goal of the study was to utilize data within scale up and acceptability frameworks, which informed the structure of the themes. | Methods (Pages 9-10) |
| 27. Software | What software, if applicable, was used to manage the data? | NVivo qualitative software, version 12 | N/A |
| 28. Participant checking | Did participants provide feedback on the ﬁndings? | No | N/A |
| *Reporting* |  |  |  |
| 29. Quotations presented | Were participant quotations presented to illustrate the themes/ﬁndings? Was each quotation identiﬁed? e.g. participant number | Yes, quotations were presented to illustrate the themes/findings. Participants were identified as working at either a ‘high’ or ‘low’ STI POCT performing clinic. | Methods (Page 8); Results |
| 30. Data and ﬁndings consistent | Was there consistency between the data presented and the ﬁndings? | Yes, there was consistency between the data presented and the findings. | Results |
| 31. Clarity of major themes | Were major themes clearly presented in the ﬁndings? | Yes, major themes were clearly presented in the Results using specific sub-sections regarding each theme (i.e., Acceptability – burden, self-efficacy, perceived effectiveness; Compatibility; and Reach). | Results |
| 32. Clarity of minor themes | Is there a description of diverse cases or discussion of minor themes? | The broader ‘Acceptability’ theme is presented under three sub-themes, or components of Acceptability: burden, self-efficacy, and perceived effectiveness. | Results |
